# Predictors of postpartum depressive symptoms: time perspective and circadian rhythm disturbances in early pregnancy.

**DOI:** 10.1192/j.eurpsy.2026.10591

**Published:** 2026-07-31

**Authors:** E. Plucińska, M. Sobol, A. Błachnio, A. Wdowiak, I. Hryhorchuk, P. Szczepaniak, J. Stasiniewicz

**Affiliations:** 1 Faculty of Psychology, University of Warsaw, Warsaw; 2 Department of Psychiatry, PZOL, Miedzybrodzie Bialskie; 3 Faculty of Social Sciences, The John Paul II Catholic University of Lublin; 4 Department of Obstetrics and Gynecology, Medical University of Lublin, Lublin; 5 Department of Obstetrics and Gynecology, ICZ Healthcare, Żywiec, Poland

## Abstract

**Introduction:**

Postpartum depression is a significant mental health disorder that can have profound and detrimental effects on both the mother and child. Early intervention, facilitated by a comprehensive understanding of the risk factors associated with this condition, is crucial for mitigating the onset of postpartum depressive symptoms.

**Objectives:**

The aim of this study was to investigate the effect of time perspective and circadian rhythm disturbances on postpartum depressive symptoms.

**Methods:**

Participants were 126 women. They were examined in the first trimester of pregnancy and during the first 3 months after delivery. At each stage, the women completed questionnaires—the Zimbardo Time Perspective Inventory, the Dark Future Scale, the Biological Rhythms Interview for Assessment in Neuropsychiatry, and the Edinburgh Postpartum Depression Scale. The participants also wore an actigraphy monitor for 1 week.

**Results:**

The results showed that fatalistic time perspective, subjectively perceived disturbances of circadian rhythms, and a tendency to focus on a negative future in the first trimester were associated with increased symptoms of postpartum depression. Moreover, the association of fatalistic and future negative perspectives with symptoms of postpartum depression was explained by the perception of disturbances of circadian rhythms. There were no significant relationships between objective and self-report measures of circadian rhythm disturbances.

**Conclusions:**

Our findings indicate that minimizing a negative time perspective during the first trimester may contribute to a reduced sense of circadian rhythm disturbances, thereby potentially alleviating postpartum depressive symptoms.

**Disclosure of Interest:**

E. Plucińska Grant / Research support from: Malgorzata Sobol reports financial support was provided by National Science Centre Poland. If there are other authors, they declare that they have no known competing financial interests or personal relationships that could have appeared to influence the work reported in this paper., M. Sobol Grant / Research support from: Grant / Research support from: Malgorzata Sobol reports financial support was provided by National Science Centre Poland. If there are other authors, they declare that they have no known competing financial interests or personal relationships that could have appeared to influence the work reported in this paper., A. Błachnio: None Declared, A. Wdowiak: None Declared, I. Hryhorchuk: None Declared, P. Szczepaniak: None Declared, J. Stasiniewicz: None Declared

